# Nicotine Pouch and E-Cigarette Use and Co-Use Among US Youths in 2023 and 2024

**DOI:** 10.1001/jamanetworkopen.2025.6739

**Published:** 2025-04-30

**Authors:** Dae-Hee Han, Alyssa F. Harlow, Richard A. Miech, Dayoung Bae, Junhan Cho, Hongying D. Dai, Steven Y. Sussman, Louisiana M. Sanchez, Leah Meza, Adam M. Leventhal

**Affiliations:** 1 Department of Population and Public Health Sciences, University of Southern California, Los Angeles; 2 Institute for Addiction Science, University of Southern California, Los Angeles; 3 USC Norris Comprehensive Cancer Center, Los Angeles, California; 4 Institute for Social Research, University of Michigan, Ann Arbor; 5 Department of Biostatistics, University of Nebraska Medical Center, Omaha

## Abstract

**Importance:**

The market sector of flavored commercial nicotine products without tobacco leaves recently expanded to nicotine pouches. Comprehensive epidemiologic analyses of patterns and trends in youth use of commercial nontobacco nicotine products are lacking.

**Objective:**

To estimate US youth nicotine pouch and e-cigarette use prevalence and associated sociodemographic variables in 2023 and 2024.

**Design, Setting, and Participants:**

This cross-sectional study used data from annual surveys conducted in school with a nationally representative sample of 10th and 12th graders in 2023 and 2024 as part of the Monitoring the Future Study.

**Exposures:**

Self-reported sex, race, ethnicity, and college plans; survey year; grade; and population density.

**Main Outcomes and Measures:**

The primary outcomes were lifetime, past-12-month, and past-30-day self-reported nicotine pouch (yes or no) and e-cigarette (yes or no) use and co-use patterns (exclusive pouch without e-cigarettes, exclusive e-cigarette without pouches, or dual use).

**Results:**

Of the 10 146 study participants (4886 [48.2%] male and 4818 [47.3%] female; 5674 [51.6%] in 10th grade and 4472 [48.4%] in 12th grade), nicotine pouch use increased from 2023 vs 2024 for lifetime use (3.0% [95% CI, 2.3%-4.0%] vs 5.4% [95% CI, 4.2%-6.8%]; risk difference [RD], 2.3% [95% CI, 1.0%-3.6%]; risk ratio [RR], 1.76 [95% CI, 1.30-2.40]), use in the past 12 months (2.4% [95% CI, 1.7%-3.2%] vs 4.6% [95% CI, 3.5%-5.9%]; RD, 2.2% [95% CI, 1.0%-3.4%]; RR, 1.95 [95% CI, 1.39-2.74]), and use in the past 30 days (1.3% [95% CI, 0.8%-1.8%] vs 2.6% [95% CI, 1.9%-3.4%]; RD, 1.3% [95% CI, 0.5%-2.1%]; RR, 2.05 [95% CI, 1.33-3.16]). E-cigarette use did not significantly change from 2023 to 2024 for lifetime use (28.5% [95% CI, 26.3%-30.7%] vs 26.7% [95% CI, 24.3%-29.3%]) and past-30-day use (13.4% [95% CI, 11.8%-15.2%] vs 11.8% [95% CI, 10.2%-13.7%]) but decreased for past-12-month use (20.0% [95% CI, 18.1%-22.0%] vs 17.6% [95% CI, 15.7%-19.7%]). From 2023 to 2024, exclusive nicotine pouch and pouch plus e-cigarette dual use generally increased, whereas exclusive e-cigarette use decreased, collectively constituting no significant change in the prevalence of any nontobacco nicotine use (using 1 or both products) across years. Demographic comparisons collapsed across 2023 to 2024 found that (1) male individuals reported higher nicotine pouch use prevalence than female individuals, whereas female individuals reported higher e-cigarette use prevalence than male individuals; (2) rural vs urban and non-Hispanic White vs Hispanic youths were more likely to use each product, with larger differences for pouches than e-cigarettes; and (3) nicotine pouch and e-cigarette use prevalence was higher among 12th vs 10th graders and those without vs with 4-year college plans.

**Conclusions and Relevance:**

In this cross-sectional study, commercial nontobacco nicotine use in US 10th and 12th graders shifted from 2023 to 2024, marked by a doubling in nicotine pouch past-30-day use, an increase in pouch plus e-cigarette dual use, and a decrease in exclusive e-cigarette use. Prioritizing surveillance, regulation, and prevention addressing pediatric nicotine pouch use warrants consideration.

## Key Points

**Question:**

What are the prevalence of, cross-year changes in, and sociodemographic variables associated with self-reported nicotine pouch and e-cigarette use and co-use among US 10th and 12th graders in 2023 and 2024?

**Findings:**

In this cross-sectional study of survey data on a nationally representative sample of 10 146 youths, nicotine pouch lifetime use (3.0% to 5.4%), use in the past 12 months (2.4% to 4.6%), and use in the past 30 days (1.3% to 2.6%) and nicotine pouch plus e-cigarette co-use significantly increased from 2023 to 2024, whereas e-cigarette use in the past 12 months declined. Pouch use was higher among 12th grade, male, rural, and non-Hispanic White youths.

**Meaning:**

These findings suggest that US youth nicotine pouch use and dual use with e-cigarettes increased from 2023 to 2024, and surveillance, regulation, and prevention efforts addressing pediatric nicotine use are warranted.

## Teaser

This cross-sectional study examines the prevalence of and sociodemographic variables associated with nicotine pouch and e-cigarette use among US youths in 2023 and 2024.

## Introduction

Prevention efforts in the US helped to mold adolescents’ perceptions of tobacco as being carcinogenic and socially undesirable, causing US youth nicotine use to decline during 1997 to 2013.^1,2^ With e-cigarettes, the first widely marketed commercial nicotine product without tobacco leaves, brands used marketing strategies to distance e-cigarettes from unfavorably viewed tobacco-containing products. E-cigarettes ultimately generated a unique nontobacco sector of the commercial nicotine market that reattracted youths to nicotine use. The commercial nontobacco nicotine market recently expanded to oral nicotine pouches, which share characteristics with e-cigarettes. Pouches are sold in fruit, candy, and mint flavors that are widely marketed on social media platforms frequented by youths.^3^ Pouches lack tobacco leaves and require no spitting, promoting perceptions that pouches can be used discreetly and go undetected by adults.^4^ Like e-cigarettes, pouches are perceived as being more socially acceptable and less harmful than cigarettes, chewing tobacco, and other tobacco-containing products.^5^

To inform current and future priorities for pediatric nicotine use prevention and policies, detailed epidemiologic analyses are needed to quantify and compare the prevalence, trends, patterns, and populations of youths who use nicotine pouches, e-cigarettes, or both products. A federal report of National Youth Tobacco Survey (NYTS) data on nontobacco commercial nicotine use stated that from 2023 to 2024, past-30-day e-cigarette use decreased and nicotine pouch use did not significantly change among US high school students.^6^ This report was accompanied by a media release^7^ that emphasized the nonsignificant increase in current pouch use and was cited in the US Food and Drug Administration (FDA) January 2025 decision to authorize marketing of 20 cinnamon, coffee, and other flavored, sweetened nicotine pouches manufactured by ZYN,^8^ the most widely sold nicotine pouch brand in the US.^9^ It may be premature to draw policy and practice implications about US youth nontobacco nicotine use given the important gaps in the NYTS reporting of pouch and e-cigarette use in 2023 and 2024,^6,10^ including no examination of co-use patterns, cross-product head-to-head demographic comparisons, and geographic and socioeconomic use disparity estimates. Additionally, NYTS reporting focused on past-30-day use changes, without assessing past-12-month use outcomes or cross-year difference tests in ever use. Past-30-day use indicators have higher specificity but lower sensitivity than other measures (eg, lifetime use) for identifying youth at risk for long-term frequent use patterns^11^ because intermittent nicotine use during adolescence commonly matures into long-term nicotine use trajectories throughout adulthood.^12^

This cross-sectional study of US adolescents analyzed the prevalence of nicotine pouch and e-cigarette use in 2023 and 2024 among 10th and 12th grade students in the Monitoring the Future (MTF) study. To comprehensively characterize the size and composition of the youth population who use nontobacco nicotine products, exclusive and dual use patterns and sociodemographic correlates of using the 2 products were estimated.

## Methods

### Sample

MTF conducts annual in-classroom self-administered surveys in nationally representative US youth samples.^13^ MTF first measured nicotine pouch use in a randomly selected one-third of 10th and 12th grade participants in 2023 (February 14 to June 2) and 2024 (February 12 to June 5). The analytic sample was composed of students with complete study outcome data (see the eFigure in Supplement 1). The study was approved by the University of Michigan institutional review board. Passive (letter sent to parents or caregivers) or active (written) informed consent was obtained, per school policy, from parents of participants younger than 18 years or from participants aged at least 18 years. This study followed the Strengthening the Reporting of Observational Studies in Epidemiology (STROBE) reporting guidelines for cross-sectional studies.

### Measures

#### Outcomes

Students completed survey items assessing nicotine e-cigarette vaping and nicotine pouch use over their lifetime, past 12 months, and past 30 days. Responses were coded dichotomously (yes vs no). For each reporting time frame, a 4-level nontobacco nicotine co-use variable was created (no use [neither nicotine pouch nor e-cigarette use], exclusive nicotine pouch use [use of pouches but not e-cigarettes], exclusive e-cigarette use [use of e-cigarettes but not pouches], and dual use [use of both pouches and e-cigarettes during respective interval]), as well as a binary any nontobacco nicotine use variable (using 1 or both vs 0 products).

#### Exposures

Participants self-reported on the survey their sex (female, male, or another sex or prefer not to answer), self-identified race and ethnicity (Hispanic, non-Hispanic Black, non-Hispanic White, other [Asian American, American Indian or Alaska Native, Native Hawaiian or Other Pacific Islander, Middle Eastern, or multiple races; responses were combined because of low frequencies]), and plan for attending a 4-year college (yes or no; a socioeconomic status indicator).^14^ Population density of school location (urban, suburban or town, and rural) and survey year were coded. Data on race and ethnicity are included in the study to describe the sample and populations who used e-cigarettes and pouches.

### Statistical Analysis

Prevalence of lifetime, past-12-month, and past-30-day use of nicotine pouches and e-cigarettes were calculated separately for each product across 2023 and 2024, and stratified by grade and sociodemographic factors. Yearly prevalence estimates were estimated for pouch and e-cigarette co-use patterns in the overall sample. Changes across survey years were tested using χ^2^ tests with Rao-Scott adjustments.^15^

Using pooled 2023 to 2024 data, modified log link Poisson regression models for binary outcomes estimated bivariable associations of survey year and each sociodemographic characteristic with nicotine pouch and e-cigarette use. Association estimates for nicotine pouch and e-cigarette use outcomes were compared head-to-head by examining overlapping 95% CIs. Sociodemographic characteristic-by-year interaction terms were tested to determine whether cross-year changes in nicotine pouch and e-cigarette use differed by sociodemographic factors. Supplementary analyses tested models including year and all sociodemographic variables as simultaneous regressors to examine statistical independence of associations.

Estimates among demographic subgroups with numbers less than 25 were deemed insufficient for calculating precise estimates and, therefore, were omitted. Analyses were weighted to calculate nationally representative estimates using R statistical software version 4.2.1 (R Project for Statistical Computing) with the survey package to account for the complex survey design. Missing data for sociodemographic variables (7% missing data rates) were addressed using multiple imputation with chained equations.^16^ Results are reported as weighted percentages, estimated total population counts, risk differences (RDs), and risk ratios (RRs) with 95% CIs. Significance was set to *P* < .05 (2-tailed) and uncorrected for multiple testing given the study’s exploratory approach.

## Results

### Descriptive Analyses

In the pooled 2023 to 2024 analytic sample, of 10 146 individuals, 5674 (51.6%) were 10th graders and 4472 (48.4%) were 12th graders. With regard to sex, 4886 (48.2%) were male, 4818 (47.3%) were female, and 442 (4.5%) reported another sex or preferred not to report sex. With regard to race and ethnicity, 2890 (32.8%) self-identified as Hispanic or Latino, 1504 (15.9%) as non-Hispanic Black, 5045 (44.1%) as non-Hispanic White, and 707 (7.3%) as another non-Hispanic race or multiracial. A total of 2820 (29.9%) lived in urban areas, 6474 (60.9%) lived in suburbs or towns, and 852 (9.2%) lived in rural areas. Finally, 2125 (23.2%) reported no plan to attend a 4-year college (Table 1).

**Table 1.** Sociodemographic Characteristics

Characteristic^b^	Participants, No. (%) [95% CI]^a^		
	Pooled 2023-2024 (N = 10 146)	2023 (n = 4963)	2024 (n = 5183)
Grade			
10th	5674 (51.6) [41.3-61.8]	2641 (52.6) [41.6-63.4]	3033 (50.6) [38.9-62.3]
12th	4472 (48.4) [38.2-58.7]	2322 (47.4) [36.6-58.4]	2150 (49.4) [37.7-61.1]
Sex			
Female	4818 (47.3) [45.5-49.0]	2364 (48.6) [46.4-50.8]	2454 (45.9) [43.5-48.4]
Male	4886 (48.2) [46.5-50.0]	2347 (46.3) [44.0-48.5]	2539 (50.2) [47.8-52.6]
Another or prefer not to answer	442 (4.5) [3.9-5.2]	252 (5.1) [4.3-6.2]	190 (3.9) [3.2-4.6]
Race and ethnicity			
Hispanic or Latino^c^	2890 (32.8) [26.8-39.4]	1376 (32.2) [25.4-39.8]	1514 (33.4) [27.3-40.0]
Non-Hispanic Black	1504 (15.9) [12.3-20.3]	654 (14.3) [10.7-18.9]	850 (17.5) [13.0-23.1]
Non-Hispanic White	5045 (44.1) [38.2-50.2]	2588 (46.4) [39.6-53.4]	2457 (41.8) [35.5-48.4]
Other^d^	707 (7.3) [5.5-9.4]	345 (7.1) [5.3-9.5]	362 (7.4) [5.3-10.2]
Population density			
Urban	2820 (29.9) [21.5-39.8]	1347 (29.9) [20.8-41.0]	1473 (29.8) [20.2-41.6]
Suburban or town	6474 (60.9) [50.8-70.1]	3249 (58.1) [47.1-68.4]	3225 (63.6) [51.9-73.8]
Rural	852 (9.2) [6.0-14.2]	367 (11.9) [7.3-18.9]	485 (6.6) [4.1-10.5]
4-y College plans			
No	2125 (23.2) [20.9-25.7]	988 (22.3) [19.6-25.2]	1137 (24.1) [21.5-27.0]
Yes	8021 (76.8) [74.3-79.1]	3975 (77.7) [74.8-80.4]	4046 (75.9) [73.0-78.5]

^a^ Column proportions were weighted to produce nationally representative estimates.

^b^ Sociodemographic characteristics, including race and ethnicity, were self-reported.

^c^ Denotes Mexican American or Chicano, Cuban American, Puerto Rican, or another Hispanic or Latino ethnicity.

^d^ Denotes Asian American, American Indian or Alaska Native, Native Hawaiian or Other Pacific Islander, Middle Eastern, or multiple races.

### Nicotine Pouch and E-Cigarette Use Trends During 2023 to 2024

Nicotine pouch use prevalence significantly increased from 2023 to 2024 for lifetime use (3.0% [95% CI, 2.3% to 4.0%] to 5.4% [95% CI, 4.2% to 6.8%]; RD, 2.3% [95% CI, 1.0% to 3.6%]; RR, 1.76 [95% CI, 1.30-2.40]), past-12-month use (2.4% [95% CI, 1.7% to 3.2%] to 4.6% [95% CI, 3.5% to 5.9%]; RD, 2.2% [95% CI, 1.0% to 3.4%]; RR, 1.95 [95% CI, 1.39-2.74]), and past-30-day use (1.3% [95% CI, 0.8% to 1.8%] to 2.6% [95% CI, 1.9% to 3.4%]; RD, 1.3% [95% CI, 0.5% to 2.1%]; RR, 2.05 [95% CI, 1.33-3.16]) (Table 2). Reductions in e-cigarette lifetime use (28.5% [95% CI, 26.3% to 30.7%] to 26.7% [95% CI, 24.3% to 29.3%]; RD, −1.8% [95% CI, −4.4% to 0.9%]) and past-30-day use (13.4% [95% CI, 11.8% to 15.2%] to 11.8% [95% CI, 10.2% to 13.7%]; RD, −1.6% [95% CI, −3.6% to 0.3%]) from 2023 to 2024 were not significant. Past-12-month e-cigarette use significantly decreased from 2023 to 2024 (20.0% [95% CI, 18.1% to 22.0%] to 17.6% [95% CI, 15.7% to 19.7%]; RD, −2.4% [95% CI, −4.6% to −0.2%]).

**Table 2.** Nicotine Pouch Use and E-Cigarette Use Prevalence in US 10th and 12th Grade Students, by Year^a^

Outcome	Students, total estimated No. (%) [95% CI]^b^		Change from 2023 to 2024	
	2023	2024	RD, % (95% CI)^c^	*P *value^d^
Nicotine pouch use				
Lifetime	227 000 (3.0) [2.3 to 4.0]	400 000 (5.4) [4.2 to 6.8]	2.3 (1.0 to 3.6)	<.001
Past 12 mo	177 000 (2.4) [1.7 to 3.2]	343 000 (4.6) [3.5 to 5.9]	2.2 (1.0 to 3.4)	<.001
Past 30 d	94 000 (1.3) [0.8 to 1.8]	192 000 (2.6) [1.9 to 3.4]	1.3 (0.5 to 2.1)	<.001
E-cigarette use				
Lifetime	2 135 000 (28.5) [26.3 to 30.7]	1 999 000 (26.7) [24.3 to 29.3]	−1.8 (−4.4 to 0.9)	.20
Past 12 mo	1 499 000 (20.0) [18.1 to 22.0]	1 319 000 (17.6) [15.7 to 19.7]	−2.4 (−4.6 to −0.2)	.04
Past 30 d	1 007 000 (13.4) [11.8 to 15.2]	884 000 (11.8) [10.2 to 13.7]	−1.6 (−3.6 to 0.3)	.11

Abbreviation: RD, risk difference.

^a^ Estimates were weighted to be representative of the overall US population of 10th and 12th grade students based on 10 146 respondents.

^b^ Total numbers of 10th and 12th grade students in the US were rounded to the nearest 1000.

^c^ The 2023 estimate was subtracted from the 2024 estimate.

^d^ *P* values were calculated from χ^2^ tests with Rao and Scott adjustment.

Table 3 depicts nicotine pouch and e-cigarette co-use pattern prevalences. An estimated 1 018 000 and 951 000 US 10th and 12th graders reported past-30-day use of these nontobacco nicotine products in 2023 and 2024, respectively. Prevalence of any nontobacco nicotine use (using 1 or both products) did not significantly change from 2023 to 2024 (lifetime use, 28.8% [95% CI, 26.6%-31.0%] to 27.4% [95% CI, 24.9%-30.0%]; past-12-month use, 20.3% [95% CI, 18.4%-22.4%] to 18.6% [95% CI, 16.6%-20.8%]; past-30-day use, 13.6% [95% CI, 11.9%-15.4%] to 12.7% [95% CI, 11.0%-14.7%]). Exclusive and dual-use patterns changed across time. Exclusive nicotine pouch use without e-cigarette co-use significantly increased from 2023 to 2024 for lifetime use (0.3% [95% CI, 0.2%-0.5%] to 0.6% [95% CI, 0.4%-1.0%]), past-12-month use (0.3% [95% CI, 0.2%-0.5%] to 1.0% [95% CI, 0.6%-1.5%]), and past-30-day use (0.2% [95% CI, 0.1%-0.3%] to 0.9% [95% CI, 0.6%-1.4%]). Exclusive e-cigarette use without pouch co-use significantly decreased from 2023 to 2024 for lifetime use (25.7% [95% CI, 23.8%-27.7%] to 22.0% [95% CI, 19.8%-24.4%]), past-12-month use (17.9% [95% CI, 16.3%-19.6%] to 14.0% [95% CI, 12.4%-15.8%]), and past-30-day use (12.3% [95% CI, 10.8%-14.0%] to 10.1% [95% CI, 8.7%-11.8%]). Dual nicotine pouch plus e-cigarette use significantly increased from 2023 to 2024 for lifetime use (2.7% [95% CI, 2.1%-3.6%] to 4.7% [95% CI, 3.7%-6.0%]) and past-12-month use (2.1% [95% CI, 1.5%-2.8%] to 3.6% [95% CI, 2.8%-4.7%]); past-30-day dual pouch plus e-cigarette use increased (1.1% [95% CI, 0.7%-1.6%] to 1.7% [95% CI, 1.2%-2.3%]) but the increase was not significant.

**Table 3.** Prevalence of Nicotine Pouch and E-Cigarette Co-Use Patterns in US 10th and 12th Grade Students, by Year^a^

Outcome	Students, total estimated No. (%) [95% CI]^b^		Change from 2023 to 2024	
	2023	2024	RD, % (95% CI)^c^	*P *value^d^
Lifetime use				
Exclusive e-cigarette use	1 930 000 (25.7) [23.8 to 27.7]	1 647 000 (22.0) [19.8 to 24.4]	−3.7 (−6.4 to −1.1)	.006
Exclusive nicotine pouch	22 000 (0.3) [0.2 to 0.5]	48 000 (0.6) [0.4 to 1.0]	0.3 (0.03 to 0.7)	.03
Dual use (pouch plus e-cigarette)	205 000 (2.7) [2.1 to 3.6]	352 000 (4.7) [3.7 to 6.0]	2.0 (0.8 to 3.2)	<.001
Any nontobacco nicotine use^e^	2 157 000 (28.8) [26.6 to 31.0]	2 047 000 (27.4) [24.9 to 30.0]	−1.4 (−4.1 to 1.3)	.31
Past-12-mo use				
Exclusive e-cigarette use	1 345 000 (17.9) [16.3 to 19.6]	1 048 000 (14.0) [12.4 to 15.8]	−3.9 (−6.0 to −1.9)	<.001
Exclusive nicotine pouch	22 000 (0.3) [0.2 to 0.5]	72 000 (1.0) [0.6 to 1.5]	0.7 (0.2 to 1.1)	<.001
Dual use (pouch plus e-cigarette)	154 000 (2.1) [1.5 to 2.8]	271 000 (3.6) [2.8 to 4.7]	1.5 (0.6 to 2.6)	.001
Any nontobacco nicotine use^e^	1 522 000 (20.3) [18.4 to 22.4]	1 391 000 (18.6) [16.6 to 20.8]	−1.7 (−3.9 to 0.5)	.13
Past-30-d use				
Exclusive e-cigarette use	924 000 (12.3) [10.8 to 14.0]	759 000 (10.1) [8.7 to 11.8]	−2.2 (−4.0 to −0.3)	.02
Exclusive nicotine pouch	11 000 (0.2) [0.1 to 0.3]	68 000 (0.9) [0.6 to 1.4]	0.7 (0.4 to 1.1)	<.001
Dual use (pouch plus e-cigarette)	83 000 (1.1) [0.7 to 1.6]	124 000 (1.7) [1.2 to 2.3]	0.6 (−0.04 to 1.1)	.07
Any nontobacco nicotine use^e^	1 018 000 (13.6) [11.9 to 15.4]	951 000 (12.7) [11.0 to 14.7]	−0.9 (−2.8 to 1.1)	.38

Abbreviation: RD, risk difference.

^a^ Estimates were weighted to be representative of the overall US population of 10th and 12th grade students based on 10 146 respondents.

^b^ Total numbers of 10th and 12th grade students in the US were rounded to the nearest 1000.

^c^ The 2023 estimate was subtracted from the 2024 estimate.

^d^ *P* values were calculated from χ^2^ test with Rao & Scott adjustment.

^e^ Denotes use of nicotine pouches, e-cigarettes, or both products.

### Differences in Sociodemographic Factors Associated With Nicotine Pouch and E-Cigarette Use

Table 4 depicts nicotine pouch and e-cigarette use prevalence by sociodemographic factors, by year. The Figure depicts cross-product comparisons of 2023 to 2024 pooled associations of year and sociodemographic variables with pouch and e-cigarette use. Female vs male youths reported lower lifetime nicotine pouch use (2023, 1.9% [95% CI, 1.3%-2.8%] vs 4.4% [95% CI, 3.2%-6.1%]; 2024, 2.7% [95% CI, 2.0%-3.6%] vs 7.8% [95% CI, 5.9%-10.4%]; pooled RR, 0.37 [95% CI, 0.28-0.50]) but higher e-cigarette use (2023, 32.7% [95% CI, 30.1%-35.4%] vs 23.1% [95% CI, 20.4%-26.0%]; 2024, 29.3% [95% CI, 26.1%-32.6%] vs 24.2% [95% CI, 21.4%-27.1%]; pooled RR, 1.31 [95% CI, 1.21-1.42]) (Figure, panel A). Hispanic vs non-Hispanic White youths had lower lifetime nicotine pouch use (2023, 1.1% [95% CI, 0.5%-2.4%] vs 5.4% [95% CI, 4.2%-6.9%]; 2024, 3.1% [95% CI, 2.0%-4.7%] vs 9.0% [95% CI, 7.1%-11.4%]; pooled RR, 0.30 [95% CI, 0.19-0.45]) but did not significantly differ in lifetime e-cigarette use (2023, 28.4% [95% CI, 25.6%-31.4%] vs 31.3% [95% CI, 28.5%-34.3%]; 2024, 25.8% [95% CI, 22.0%-30.1%] vs 29.3% [95% CI, 25.9%-33.0%]; pooled RR, 0.89 [95% CI, 0.78-1.02]). Rural vs urban youths reported higher lifetime use of each product, with higher RRs for nicotine pouches (2023, 7.5% [95% CI, 4.6%-12.1%] vs 1.6% [95% CI, 0.8%-3.3%]; 2024, 11.2% [95% CI, 8.2%-15.0%] vs 3.0% [95% CI, 1.8%-5.0%]; pooled RR, 3.89 [95% CI, 2.31-6.54]) than e-cigarettes (2023, 35.9% [95% CI, 29.6%-42.7%] vs 23.7% [95% CI, 19.6%-28.3%]; 2024, 34.4% [95% CI, 28.4%-41.0%] vs 22.2% [95% CI, 17.9%-27.2%]; RR, 1.54 [95% CI, 1.26-1.88]). Lifetime nicotine pouch and e-cigarette use risks were each similarly higher for 12th vs 10th graders and suburban vs urban youths, but were each similarly lower in youths with vs without 4-year college plans. Sociodemographic variable results for past-12-month and past-30-day nicotine pouch and e-cigarette use largely paralleled lifetime use results (Figure, panels B and C).

**Table 4.** Nicotine Pouch and E-Cigarette Use Prevalence in Each Year, by Sociodemographic Characteristics

Characteristic^b^	Use prevalence, % (95% CI)^a^					
	Lifetime		Past 12 mo		Past 30 d	
	2023	2024	2023	2024	2023	2024
Nicotine pouch use						
Grade						
10th	2.6 (1.7-3.8)	4.0 (2.9-5.4)	1.9 (1.2-2.7)	3.3 (2.4-4.7)	1.1 (0.6-1.9)	1.7 (1.2-2.4)
12th	3.6 (2.4-5.3)	6.8 (4.7-9.6)	2.9 (1.9-4.5)	5.9 (4.1-8.4)	1.4 (0.8-2.5)	3.4 (2.3-5.1)
Sex						
Female	1.9 (1.3-2.8)	2.7 (2.0-3.6)	1.5 (0.9-2.4)	1.8 (1.3-2.6)	0.5 (0.2-1.2)	0.9 (0.5-1.4)
Male	4.4 (3.2-6.1)	7.8 (5.9-10.4)	3.4 (2.4-4.9)	7.2 (5.4-9.5)	2.1 (1.4-3.3)	4.3 (3.1-5.8)
Another^c^	NA^d^	NA^d^	NA^d^	NA^d^	NA^d^	NA^d^
Race and ethnicity						
Hispanic or Latino^e^	1.1 (0.5-2.4)	3.1 (2.0-4.7)	0.4 (0.2-0.9)	2.8 (1.7-4.4)	0.2 (0.1-0.6)	1.1 (0.6-1.9)
Non-Hispanic Black	NA^d^	NA^d^	NA^d^	NA^d^	NA^d^	NA^d^
Non-Hispanic White	5.4 (4.2-6.9)	9.0 (7.1-11.4)	4.7 (3.6-6.1)	7.8 (6.0-10.0)	2.4 (1.6-3.6)	4.6 (3.3-6.4)
Other^f^	NA^d^	NA^d^	NA^d^	NA^d^	NA^d^	NA^d^
Population density						
Urban	1.6 (0.8-3.3)	3.0 (1.8-5.0)	0.9 (0.5-1.9)	2.8 (1.6-4.7)	0.8 (0.4-1.6)	1.3 (0.8-2.2)
Suburban or town	2.9 (2.0-4.0)	5.9 (4.2-8.1)	2.1 (1.5-3.0)	4.9 (3.5-7.0)	0.9 (0.5-1.4)	2.6 (1.7-4.0)
Rural	7.5 (4.6-12.1)	11.2 (8.2-15.0)	7.2 (4.3-11.8)	9.7 (6.8-13.7)	4.4 (2.4-7.9)	7.7 (5.2-11.3)
4-y College plans						
No	5.4 (3.7-7.9)	8.0 (5.8-10.8)	3.9 (2.4-6.2)	6.9 (5.1-9.3)	2.5 (1.3-4.8)	3.9 (2.8-5.6)
Yes	2.3 (1.7-3.2)	4.5 (3.5-5.8)	1.9 (1.4-2.7)	3.9 (2.9-5.1)	0.9 (0.6-1.4)	2.1 (1.5-3.0)
E-cigarette use						
Grade						
10th	25.0 (22.4-27.8)	22.6 (19.8-25.7)	16.9 (14.8-19.1)	15.1 (13.0-17.5)	10.9 (9.5-12.4)	9.5 (8.0-11.3)
12th	32.3 (28.6-36.1)	30.9 (27.4-34.6)	23.4 (20.1-27.1)	20.2 (17.2-23.5)	16.2 (13.1-20.0)	14.2 (11.6-17.3)
Sex						
Female	32.7 (30.1-35.4)	29.3 (26.1-32.6)	22.9 (20.5-25.5)	19.9 (17.5-22.5)	14.8 (12.7-17.1)	13.5 (11.7-15.5)
Male	23.1 (20.4-26.0)	24.2 (21.4-27.1)	16.1 (13.8-18.8)	15.3 (12.9-18.0)	11.2 (9.2-13.5)	10.0 (7.9-12.5)
Another^c^	36.7 (29.5-44.5)	29.1 (22.6-36.7)	27.3 (20.2-35.8)	20.9 (14.0-30.2)	21.1 (15.0-28.8)	15.3 (9.3-24.1)
Race and ethnicity						
Hispanic or Latino^e^	28.4 (25.6-31.4)	25.8 (22.0-30.1)	17.1 (14.3-20.3)	15.9 (12.7-19.6)	11.1 (8.9-13.7)	10.3 (8.0-13.1)
Non-Hispanic Black	25.7 (20.4-31.9)	25.1 (21.6-29.1)	18.0 (13.9-23.0)	15.2 (12.0-19.0)	11.2 (8.3-15.1)	9.6 (7.4-12.5)
Non-Hispanic White	31.3 (28.5-34.3)	29.3 (25.9-33.0)	23.9 (21.4-26.5)	20.9 (18.3-23.8)	16.5 (14.2-18.9)	14.6 (12.4-17.0)
Other^f^	15.9 (11.0-22.6)	19.6 (13.2-28.2)	11.8 (4.5-18.3)	12.5 (8.0-19.0)	8.5 (4.6-15.2)	8.3 (4.9-13.8)
Population density						
Urban	23.7 (19.6-28.3)	22.2 (17.9-27.2)	16.4 (13.3-20.1)	13.7 (10.5-17.8)	8.7 (6.6-11.5)	8.7 (6.0-12.4)
Suburban or town	29.4 (26.8-32.1)	28.0 (25.1-31.1)	20.4 (17.9-23.2)	18.7 (16.4-21.2)	14.6 (12.4-17.1)	12.4 (10.4-14.7)
Rural	35.9 (29.6-42.7)	34.4 (28.4-41.0)	26.8 (21.5-32.8)	25.0 (20.8-29.8)	19.5 (14.3-26.0)	20.0 (16.6-23.9)
4-y College plans						
No	36.9 (32.7-41.2)	35.9 (32.5-39.4)	25.9 (21.8-30.4)	24.4 (21.3-27.8)	20.6 (16.7-25.0)	18.8 (16.0-21.9)
Yes	26.1 (23.9-28.3)	23.8 (21.3-26.5)	18.3 (16.4-20.4)	15.5 (13.6-17.5)	11.4 (9.7-13.2)	9.6 (8.1-11.3)

Abbreviation: NA, not available.

^a^ Estimates are weighted column percentages.

^b^ Sociodemographic characteristics, including race and ethnicity, were self-reported on the survey.

^c^ Denotes another sex or prefer not to answer.

^d^ Estimates are not presented for demographic group because the cell size is less than 25.

^e^ Refers to Mexican American or Chicano, Cuban American, Puerto Rican, and other Hispanic or Latino.

^f^ Refers to Asian American, American Indian or Alaska Native, Native Hawaiian or Other Pacific Islander, Middle Eastern, or multiple races.

**Figure.**
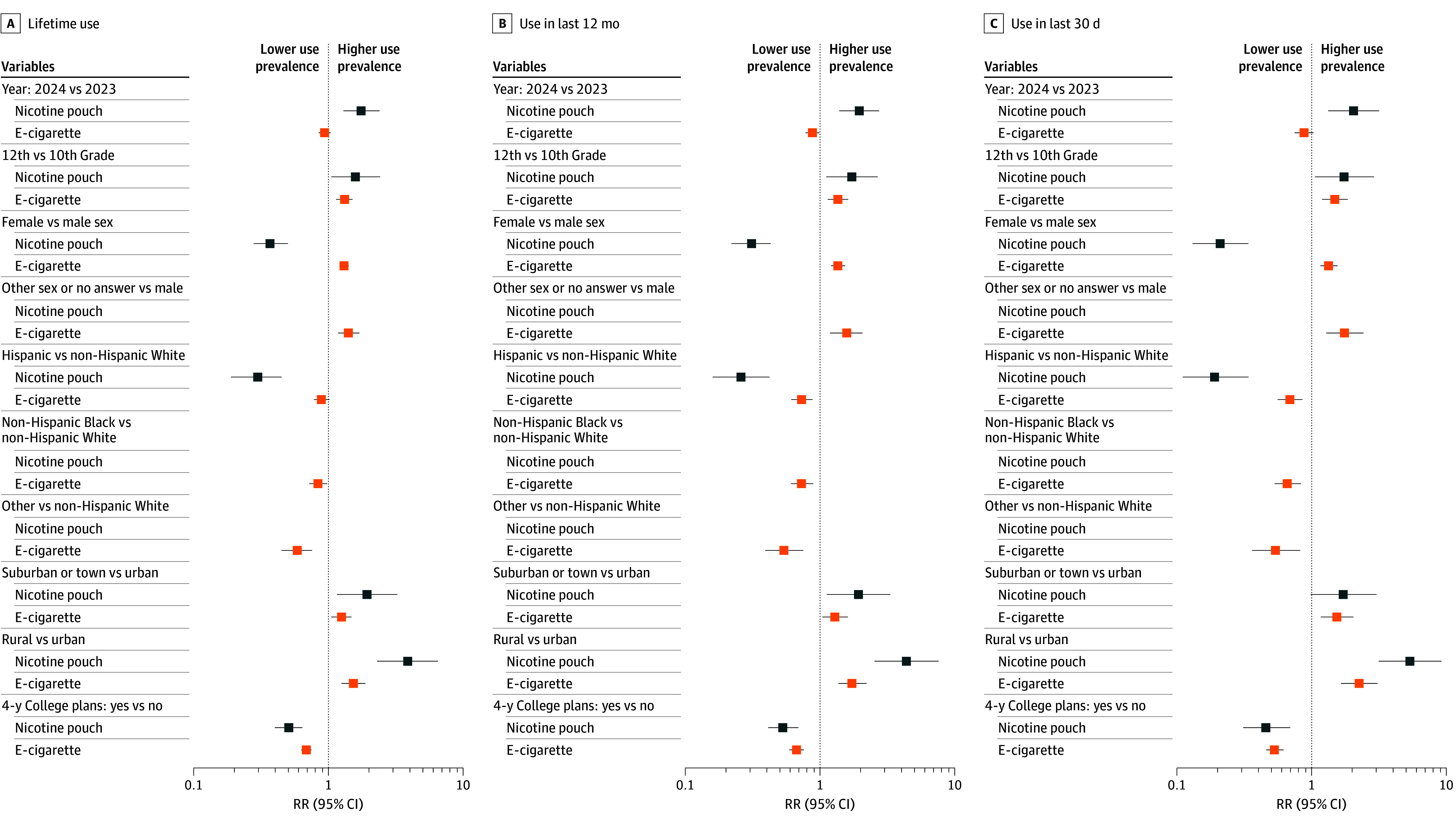
Association of Year and Sociodemographic Characteristics With Nicotine Pouch and E-Cigarette Use Risk ratios (RRs) and 95% CIs for lifetime use (A), past-12-month use (B), and past-30-day use (C) are shown.

For sociodemographic variables with frequencies high enough to estimate e-cigarette but not pouch use, e-cigarette use prevalence was lower in youths who were non-Hispanic Black or another non-Hispanic race than non-Hispanic White. E-cigarette use was higher in youths who reported being another sex besides male or female or declined to report their sex compared with male youths (Figure). Estimates from supplementary models mutually adjusting for sociodemographic characteristics largely coincided with the primary unadjusted analyses, with the exception that inverse associations of 4-year college plans with past-12-month and past-30-day nicotine pouch use became nonsignificant after adjustment (eTable 1 in Supplement 1).

Sociodemographic factor-by-year interactions were not significant (eTable 2 in Supplement 1), providing no evidence that the rate of change over 2023 to 2024 varied by sociodemographic subpopulations, with one exception. Changes in past-12-month nicotine pouch use from 2023 to 2024 varied between Hispanic and non-Hispanic White youths, owing to Hispanic youths’ low 2023 base rate (0.4%) that then increased 7-fold by 2024 (2.8%) compared with non-Hispanic White youths’ 2023 base rate (4.7%) that increased 1.7-fold by 2024 (7.8%).

## Discussion

This cross-sectional study of US adolescents found that commercial nontobacco nicotine use among 10th and 12th graders shifted from 2023 to 2024, marked by 4 findings. First, nicotine pouch use prevalence increased 1.8-fold for lifetime use, 2.0-fold for past-12-month use, and 2.1-fold for past-30-day use from 2023 to 2024. Second, past-12-month nicotine vaping decreased between 2023 and 2024. Third, diverging nicotine pouch and vaping trends during 2023 to 2024 combined led to no net change in the overall percentage of nontobacco nicotine use among US youth, but pouch plus e-cigarette dual use increased. Fourth, the sociodemographic profiles of US youths who use nicotine pouches differ from those who use e-cigarettes, particularly with respect to sex: female youths were more likely to vape and less likely to use pouches than male youths.

For more than a decade, e-cigarettes solely occupied a niche within the market as the only commercial nontobacco nicotine product with a unique combination of youth-appealing features capable of spurring an epidemic (eg, assorted flavors, concealability, social media marketing, no tobacco leaves, and low perceived harm). Declines in e-cigarette use following the youth nicotine vaping epidemic’s 2019 peak were among the largest reductions recorded in adolescent drug use epidemiology.^2^ After large decreases following the COVID-19 outbreak and further reductions in nicotine vaping during 2022 and 2023,^2^ this study observed that youth e-cigarette use trends continued downward. However, the rate of decline from 2023 to 2024 was slower than in previous years and concentrated among those who vape nicotine without pouch co-use.

Increasing nicotine pouch use prevalence from 2023 to 2024 observed in this study generalized across lifetime, past-12-month, and past-30-day use outcomes, indicating that increasing trends are not merely attributable to teens who tried and discontinued using pouches. Increasing youth nicotine pouch use coincides with 2024 sales increases of ZYN,^9^ which was the most widely used nicotine pouch brand among US youth in prior research.^17^ Because pouches deliver nicotine doses that may be addictive^18^ and possess other youth-attracting features (eg, flavors, concealability, or perceived dissociation from other nicotine products that contain tobacco leaves), it is plausible that the burgeoning nicotine pouch marketing over 2023 to 2024 translated to more youth users.

At first glance, federal reporting of NYTS nontobacco nicotine use trends^8,10^ and this study’s MTF evidence do not entirely align. However, careful inspection reveals appreciable concordance across the 2 studies’ estimates. Nicotine pouch use percentages among NYTS high school students were 3.1% (95% CI, 2.4%-4.0%) in 2023 and 4.7% (95% CI, 4.0%-5.6%) in 2024 for ever use (cross-year difference test not reported)^10,19^ and 1.7% (95% CI, 1.2%-2.5%) and 2.4% (95% CI, 2.0%-2.9%) for past-30-day use (cross-year increase not statistically significant).^6,10^ The 95% CIs overlap between the NYTS pouch use estimates and MTF’s corresponding estimates.^6,10,19^ The past-30-day e-cigarette use reduction from 2023 to 2024 was statistically significant in NYTS high school students^6^ but not significant in the present study. The current study may have had insufficient statistical power to detect small cross-year RRs because analyses were limited to the subset of MTF respondents who received pouch questions. Analyses among all MTF respondents found small cross-year reductions in e-cigarette use from 2023 to 2024 that were statistically significant for 10th graders but not significant for 12th graders.^2^

Diverging youth nicotine pouch and e-cigarette use trends appeared to cancel one another out, resulting in no total change in the number of US youths using these nontobacco nicotine products. An estimated 1 018 000 and 951 000 US 10th and 12th graders reported past-30-day use of these nontobacco nicotine products in 2023 and 2024, respectively. Similar trends were observed for lifetime and past-12-month use. Because MTF includes only 10th and 12th grades by design, these estimates reflect approximately one-half of the overall US high school student population and should be interpreted as such.

Exclusive nicotine pouch use without vaping nicotine and pouch plus e-cigarette dual use each significantly increased across years. Yet, exclusive pouch use base rates remained low in 2024, suggesting that few youths initiate use of pouches before e-cigarettes. Because nicotine pouches are diminutive and do not require spitting, youths can discreetly place pouches between their lip and gums in virtually any situation. Discretion and the capacity for use where other nicotine and tobacco products are not permitted are commonly reported reasons for using nicotine pouches among young people.^4,5^ Evidence is lacking on whether dual pouch plus e-cigarette use increases total nicotine exposure and addiction risk in the same manner that poly-use of e-cigarettes with other tobacco products has been previously implicated in nicotine addiction.^20^

The typical nicotine pouch–using youth was male, non-Hispanic White, rural, and did not plan to attend a 4-year college, which mirrors the demographic targeted in industry marketing of smokeless chewing tobacco, snuff, and dip.^21,22,23,24,25,26^ By contrast, youths who vaped nicotine were overrepresented by female youths, which departs from estimates of higher vaping in male than female youths in prior years when e-cigarettes were newer on the market and predominately sold as large tank-style devices.^27^ Female youths are more likely than male youths to prefer smaller disposable and/or pod-style electronic devices, like those that have saturated the e-cigarette market in recent years.^28,29^ Changes in nicotine pouch and e-cigarette use over 2023 and 2024 largely did not significantly differ across sociodemographic groups, suggesting that increasing nicotine pouch use may generalize to various youth demographics. Further monitoring of whether the demographic profiles of youth who use nicotine pouches remain or shift toward other populations will be important for future surveillance of health disparities.

The FDA authorized marketing of 20 ZYN flavored nicotine pouch products in January 2025, citing NYTS’s non–statistically significant past-30-day pouch use increase as supporting rationale for the decision, with the caveat that FDA could rescind authorization if there is a substantial increase in youth initiation.^8^ Meanwhile, dozens of other nicotine pouch brands are being illegally marketed and could be subject to federal enforcement if deemed a public health priority. Although the current evidence of increasing pouch use in the overall US 10th and 12th grade population heightens awareness of this issue, further surveillance and research on nicotine pouch harms will be needed to understand the public health impact of the population-wide estimates reported here. Regardless, pouch use among rural youths appears to have reached appreciable levels based on this study’s findings. Substantial nicotine exposure to the adolescent brain could interfere with development of neurocircuitry implicated in attention and mood regulation and cause addiction.^30^ Buccal nicotine absorption from pouches can be substantial but is less immediate than pulmonary delivery through inhaled products, like cigarettes.^31^ Some nicotine pouch products contain numerous potentially toxic chemicals (eg, acetaldehyde, nitrite, metals, methyl eugenol, and benzophenone), but the types of toxins and their amounts vary across brands and have unclear clinical importance.^3^ A small convenience sample study of adults found moderate levels of nicotine dependence and appreciable rates of self-reported mouth lesions and nausea from using pouches.^32^ Data on nicotine pouch health impacts from large, representative samples are lacking.

### Limitations

This study has limitations that should be mentioned. First, recall and measurement errors cannot be ruled out. Second, owing to small samples for some demographic subgroups, nicotine pouch use could not be estimated with sufficient precision in these populations. After 2024, nicotine pouch use questions will be administered to more than one-third of MTF respondents, which will allow larger samples for detailed estimates and cross-year analyses stratified by grade. Third, a cautious interpretation is necessary when generalizing the findings to all adolescents because the sample comprised solely 10th and 12th graders in school during data collection. Fourth, this study focused on 2 of the newest commercial nicotine products, which share similarities, including their lack of tobacco leaves, flavors, and marketing. Other tobacco-containing products with well-known detrimental health effects were not examined and should be studied in future investigations alongside pouches and e-cigarettes.

## Conclusions

In this cross-sectional study of US adolescents, commercial nontobacco nicotine product use among 10th and 12th graders shifted from 2023 to 2024, marked by increasing nicotine pouch use and pouch plus e-cigarette dual use and declining exclusive e-cigarette use. Nicotine pouch use was disproportionately represented by male, non-Hispanic White, and rural youths pooled across years, but the extent of changes in nicotine pouch or e-cigarette use from 2023 to 2024 largely did not differ across sociodemographic strata. Expanding regulatory protections, public health campaigns, clinical assessment, surveillance, and prevention programs addressing youth nicotine pouch use warrant consideration to improve pediatric population health.

## Notes

Supplement 1. **eTable 1.** Adjusted Risk Ratio for Association of Sociodemographic Characteristics With Outcomes **eTable 2.** P-Values From Unadjusted Model Omnibus Interaction Test (Sociodemographic Characteristics × Year) **eFigure.** Study Accrual Flowchart

Supplement 2.
Data Sharing Statement
