# Supplementary material for: Nicotine Pouch and E-Cigarette Use and Co-Use Among US Youths in 2023 and 2024
Source: JAMA Netw Open. 2025 Apr 30;8(4):e256739. doi: 10.1001/jamanetworkopen.2025.6739 (PMC12044512; doi:10.1001/jamanetworkopen.2025.6739)
Supplement: Supplement 1. — eTable 1. Adjusted Risk Ratio for Association of Sociodemographic Characteristics With Outcomes eTable 2. P-Values From Unadjusted Model Omnibus Interaction Test (Sociodemographic Characteristics × Year) eFigure. Study Accrual Flowchart [file jamanetwopen-e256739-s001.pdf]

# Supplemental Online Content

Han DH, Harlow AF, Miech RA, et al. Nicotine pouch and e-cigarette use and co-use among US youths in 2023 and 2024. *JAMA Netw Open*. 2025;8(4):e256739. doi:10.1001/jamanetworkopen.2025.6739

**eTable 1.** Adjusted Risk Ratio for Association of Sociodemographic Characteristics With Outcomes

**eTable 2.** P-Values From Unadjusted Model Omnibus Interaction Test (Sociodemographic Characteristics × Year)

**eFigure.** Study Accrual Flowchart

This supplemental material has been provided by the authors to give readers additional information about their work.

© 2025 Han DH et al. *JAMA Network Open*.

**eTable 1. Adjusted Risk Ratio for Association of Sociodemographic Characteristics With Outcomes[a]**

| Characteristic[b] | Nicotine pouch use, risk ratio (95% CI) | | | E-cigarette use, risk ratio (95% CI) | | |
|---|---|---|---|---|---|---|
| | Lifetime | Past 12-month | Past 30-day | Lifetime | Past 12-month | Past 30-day |
| **Year** | | | | | | |
| 2023 | Reference | Reference | Reference | Reference | Reference | Reference |
| 2024 | 1.85 (1.43, 2.39) | 2.08 (1.59, 2.71) | 2.24 (1.57, 3.19) | 0.95 (0.88, 1.03) | 0.90 (0.81, 1.00) | 0.90 (0.79, 1.03) |
| **Grade** | | | | | | |
| 10th | Reference | Reference | Reference | Reference | Reference | Reference |
| 12th | 1.48 (1.07, 2.04) | 1.60 (1.14, 2.23) | 1.65 (1.11, 2.44) | 1.28 (1.13, 1.44) | 1.31 (1.13, 1.52) | 1.41 (1.19, 1.68) |
| **Sex** | | | | | | |
| Male | Reference | Reference | Reference | Reference | Reference | Reference |
| Female | 0.40 (0.30, 0.53) | 0.33 (0.24, 0.45) | 0.23 (0.14, 0.36) | 1.40 (1.29, 1.51) | 1.46 (1.30, 1.63) | 1.49 (1.29, 1.71) |
| Other/prefer not to answer | NA[e] | NA[e] | NA[e] | 1.47 (1.21, 1.78) | 1.64 (1.22, 2.21) | 1.87 (1.34, 2.62) |
| **Race/ethnicity** | | | | | | |
| Non-Hispanic White | Reference | Reference | Reference | Reference | Reference | Reference |
| Hispanic/Latino[c] | 0.33 (0.21, 0.53) | 0.29 (0.18, 0.49) | 0.23 (0.12, 0.43) | 0.93 (0.80, 1.09) | 0.77 (0.62, 0.96) | 0.75 (0.58, 0.97) |
| Non-Hispanic Black | NA[e] | NA[e] | NA[e] | 0.90 (0.77, 1.06) | 0.80 (0.66, 0.97) | 0.76 (0.61, 0.95) |
| Other[d] | NA[e] | NA[e] | NA[e] | 0.65 (0.52, 0.82) | 0.61 (0.46, 0.81) | 0.66 (0.45, 0.95) |
| **Population density** | | | | | | |
| Urban | Reference | Reference | Reference | Reference | Reference | Reference |
| Suburban/town | 1.41 (0.86, 2.31) | 1.37 (0.84, 2.26) | 1.21 (0.70, 2.07) | 1.16 (0.99, 1.37) | 1.17 (0.96, 1.44) | 1.36 (1.03, 1.80) |
| Rural | 2.26 (1.34, 3.80) | 2.52 (1.19, 4.26) | 3.01 (1.70, 5.33) | 1.34 (1.10, 1.63) | 1.37 (1.09, 1.73) | 1.69 (1.24, 2.28) |
| **4-year college plan** | | | | | | |
| No | Reference | Reference | Reference | Reference | Reference | Reference |
| Yes | 0.73 (0.57, 0.92) | 0.82 (0.63, 1.06) | 0.77 (0.51, 1.16) | 0.69 (0.63, 0.75) | 0.68 (0.60, 0.76) | 0.55 (0.47, 0.64) |

Abbreviations: CI = confidence interval.

[a] The model simultaneously tested for all sociodemographic correlates in Table 1.
[b] Sociodemographic characteristics, including race and ethnicity, were self-reported. CI = confidence interval.
[c] Mexican American/Chicano, Cuban American, Puerto Rican, other Hispanic or Latino.
[d] Asian American, American Indian or Alaska Native, Native Hawaiian or other Pacific Islander, or Middle Eastern.
[e] Estimate not presented for demographic group because cell size <25.

© 2025 Han DH et al. *JAMA Network Open*.

**eTable 2. P-Values From Unadjusted Model Omnibus Interaction Test (Sociodemographic Characteristics × Year)**

| Characteristic[a] | Nicotine pouch use | | | E-cigarette use | | |
|---|---|---|---|---|---|---|
| | Lifetime | Past-12-month | Past-30-day | Lifetime | Past-12-month | Past-30-day |
| Grade | 0.74 | 0.93 | 0.35 | 0.56 | 0.75 | 0.97 |
| Sex | 0.12 | 0.10 | 0.87 | 0.49 | 0.96 | 0.73 |
| Race/ethnicity[b] | 0.18 | <.01 | 0.08 | 0.41 | 0.78 | 0.98 |
| Population density | 0.45 | 0.08 | 0.32 | 0.99 | 0.99 | 0.54 |
| College plans | 0.30 | 0.65 | 0.35 | 0.13 | 0.19 | 0.59 |

[a] Sociodemographic characteristics, including race and ethnicity, were self-reported.

[b] The models did not include other sex/prefer not to answer and non-Hispanic Black and other race/ethnicity due to too few cases of outcome with correlates.

© 2025 Han DH et al. *JAMA Network Open*.

**eFigure. Study Accrual Flowchart**

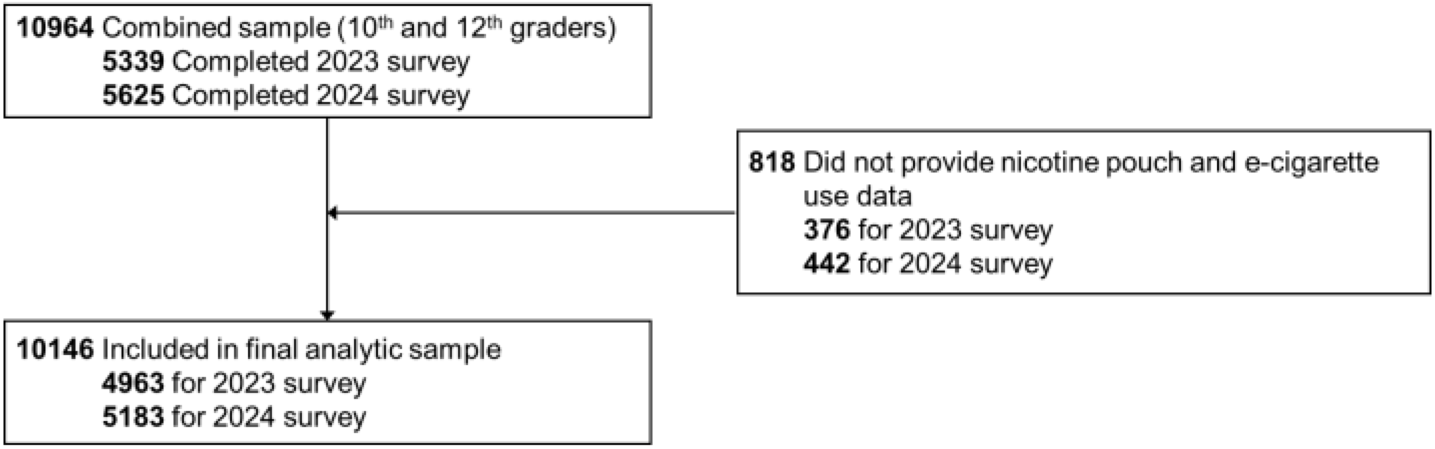


© 2025 Han DH et al. *JAMA Network Open*.
