# Supplementary material for: Nicotine Pouch and E-Cigarette Use and Co-Use Among US Youths in 2023 and 2024
Source: JAMA Netw Open. 2025 Apr 30;8(4):e256739. doi: 10.1001/jamanetworkopen.2025.6739 (PMC12044512; doi:10.1001/jamanetworkopen.2025.6739)
Supplement: Supplement 2. — Data Sharing Statement [file jamanetwopen-e256739-s002.pdf]

Data Sharing Statement

Han. Nicotine Pouch and E-Cigarette Use and Co-Use Among US Youths in 2023 and 2024. *JAMA Netw Open*. Published April 30, 2025. doi:10.1001/jamanetworkopen.2025.6739

Data
Data available: No

Additional Information
Explanation for why data not available: Data are not available immediately. The US Monitoring the Future makes data publicly available; however, there is usually an 18-month lag between data collection and making the data publicly downloadable. Because the data are currently restricted until then, there will be a delay. If a specific request for the data is made before then, requests to Dr. Miech who is the Director of the Monitoring the Future Study should be made.
